# Secondary Ion–Ice Chemistry in UV-Irradiated Oxygen Ice: New Astrochemical Models Including Bulk Ionic Species

**DOI:** 10.1021/acsearthspacechem.6c00036

**Published:** 2026-06-24

**Authors:** Kristen Darnell, Daniel Lopez-Sanders, Deaton Warren, River-Allen Carroll, Emma Stanley, Liton Majumdar, Paola Caselli, Christopher R. Arumainayagam, Christopher N. Shingledecker

**Affiliations:** † Department of Astronomy, 7161 San Jose State University, San Jose, California 95192-0106, United States; ‡ Department of Physics and Astronomy, National Radio Astronomy Observatory, Charlottesville, Virginia 22903, United States; § Department of Chemistry, 4532 Virginia Military Institute, Lexington, Virginia 24450, United States; ∥ Exoplanets and Planetary Formation Group, School of Earth and Planetary Sciences, 193155 National Institute of Science Education and Research, Jatni 752050, Odisha, India; ⊥ Homi Bhabha National Institute, Training School Complex, Anushaktinagar, Mumbai 400094, India; # Center for Astrochemical Studies, Max Planck Institute for Extraterrestrial Physics, Garching 85741, Germany; ∇ Department of Chemistry, 317288 Wellesley College, Wellesley, Massachusetts 02481, United States

**Keywords:** astrochemistry, cosmic rays, UV photons, photochemistry, ions

## Abstract

Although energetic and nonenergetic processing of interstellar dust grain surfaces and ice is thought to be one of the dominant mechanisms for the extraterrestrial synthesis of prebiotic molecules, the role that “ion–ice” chemistry (bulk ion–neutral and ion–ion reactions within the dust-grain ice mantle) plays is still a largely unexplored topic. In this work, we present the results of our study to investigate the astrochemical importance of ions produced within dust grain ice mantles via irradiation by VUV photons, though the methods are applicable to other kinds of ionizing radiation. To this end, we present new models of the chemical evolution of a pure oxygen ice irradiated by UV photons, using a chemical network that explicitly includes bulk reactions involving secondary ions. A comparison of our calculations with previous data suggests that ion–neutral and ion–ion reactions may play a critical role in understanding the chemistry of interstellar ice.

## Graphical abstract

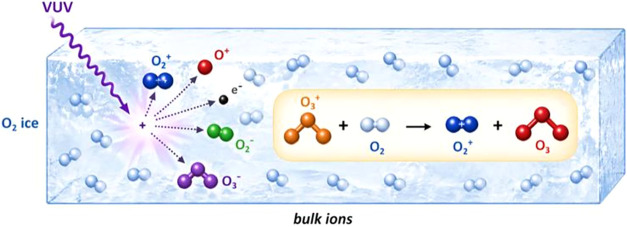

## 1 Introduction

It has now been more than half a century since the publication of the pioneering work of Herbst and Klemperer,^1^ which arguably marked the genesis of the field of astrochemical modeling. A number of key predictions from that work have since been verified. Among these was the claim that gas-phase ion-neutral reactions, beginning with those involving H_3_^+^, were essential to the formation of polyatomic molecules in interstellar environments. These reactions are often both barrierless and exothermic, making them efficient even in dense molecular clouds, which are typically around 10 K (see the review by Ceccarelli et al.^2^ and references therein for more details). In addition, especially for cases where the neutral reactant is polar, the long-range forces involved mean that such reactions can have a negative temperature dependence^3^ and rate coefficients significantly larger than the ∼10^–9^ cm^3^ s^–1^ predicted by the Langevin equation.^4^

Just as ions are critical for the formation of polyatomic molecules in the gas phase, so too are interstellar dust grains. In interstellar environments, dust acts as a quasi-catalyst for chemical reactions. In diffuse and translucent molecular clouds, the surfaces of the bare grains act as important reaction sites, especially for the formation of H_2_.^5–8^ So too are the ice mantles which form on dust grains in dense molecular clouds. Our understanding of the importance of dust-grain ice mantles in explaining the observed chemical complexity of interstellar environments has grown over the history of astrochemistry. Grain-related processes are now thought to be critical in explaining the presence of terrestrial-like saturated or semisaturated organic species, including “prebiotic” molecules which may be implicated in the origins of life on Earth and possibly elsewhere.^2^ Nevertheless, the addition of surface and bulk ice-mantle grain chemistry, though doubtless a more accurate representation of chemistry in the real interstellar medium, remains an active area of research.

The unfortunate combination of the importance of grain-related processes in the interstellar medium (ISM), combined with a comparative lack of knowledge and data regarding relevant processes has led one astrochemical modeler to make the now well-known jest that interstellar grain chemistry represents the “last refuge of the scoundrel.^9^” Here, we will highlight just a few of the current areas of uncertainty that pose challenges for ice modeling, generally. First, there exist substantial uncertainties regarding the physisorption binding energies of most of the hundreds of species usually included in grain-chemical networks.^10–12^ These binding energies, which have a range of values depending on many factors, one example being the local surface morphology, are typically approximated with a single value, with some very recent exceptions.^13,14^ Moreover, though it is now well established both via experiments and theory that grain and ice-mantle surface chemistry is dominated by diffusive Langmuir–Hinshelwood type reactions,^15–18^ whether and how chemistry within the ice mantle occurs at low temperatures has been the subject of considerable recent work. There is a growing consensus that nondiffusive processes almost certainly play a key role.^16,19–23^ Finally, and most pertinent for this work, the chemical networks used for simulating interstellar grain chemistry have historically included only neutral species.

That ion-ice processes can occur efficiently under interstellar conditions was suggested in the pioneering theoretical study of Woon,^24^ who found that cations such as HCO^+^ and CH_3_^+^ were able to react barrierlessly with water molecules in ice via pathways that could lead to formic acid and methanol, two astrochemically relevant species (see also Woon^25^). The reaction between low-energy CH_3_^+^ cations and water ice was investigated in detail by Nakai et al.,^26^ who confirmed experimentally that the reaction suggested by Woon^24^ does indeed occur under interstellar conditions. In that work, astrochemical models using the code of Furuya et al.^27^ found that the CH_3_^+^-ice reaction was competitive at the edges of the simulated molecular cloud where the visual extinction was low, and calculated enhanced abundances of gas-phase CH_3_^+^.

The adsorption of gas-phase cations undoubtedly also occurs in real interstellar environments. The interaction between gas-phase cations and ice surfaces was the subject of a recent modeling study by Cui and Herbst,^28^ who found that such reactions could be efficient in forming complex species. However, there exist other mechanisms for driving ion-ice chemistry.

While externally produced energetic photons are quickly extinguished in molecular clouds, galactic cosmic rays reach even the centers of most dense molecular clouds,^29^ and are an important source of continuous ice processing in the ISM.^30,31^ The essential role of cosmic rays to astrochemistry has been recognized from the outset of astrochemical modeling in the first model of Herbst and Klemperer.^1^ They noted that cosmic rays drive the formation of H_3_^+^ via collisions with H_2_, which also generate an internal flux of UV photons. These photons can further ionize gaseous species in molecular clouds.^32^ Direct cosmic ray collisions with dust grains and their ice mantles similarly result in the formation of charged species within a roughly cylindrical region known as the “track”^33^ which forms around the path of the cosmic ray through the solid. Bombardment by internal UV photons represents yet another means of ionizing,^34^ albeit with a lower efficiency than with cosmic rays.^35^ Although some fraction of the charged species produced within ices undoubtedly undergo rapid neutralization by recombination,^36,37^ the remainder may participate in an internal ion-ice chemistry whose importance may be comparable to that known to occur in the gas-phase. The ionization of an ice-mantle species creates, in addition to the atomic or molecular ion, a secondary electron, which can subsequently multiply the chemical effects. When such secondary electrons lose sufficient energy through collisions with bulk species, they can become “low-energy” electrons (<20 eV), which can have significant effects of their own.^35,38–40^

In this work, we present the first computational study of secondary ion-ice chemistry driven by the UV photon (<11.3 eV) bombardment of a cosmic ice analogue of pure molecular oxygen, following the experimental work of Gerakines et al.^41^ Though galactic cosmic rays are indeed the ultimate drivers of ion chemistry (both gas and solid phase) in dense molecular clouds, they generate an internal flux of VUV photons which trigger broadly similar reactions as the cosmic rays themselves. It is these VUV photons which we simulate in this work, following the laboratory conditions of Gerakines et al.^41^ The key distinction between the present work and all our previous implementations of this modeling approach^34,36^ is our relaxation of the requirement that ionization is always immediately followed by charge recombination, such that in previous studies, ionic species were never present in the simulated ice. Here, for the first time, we allow ionization to persist, yielding long-lived ionic species that participate in a network of ion–neutral, ion–ion, and dissociative recombination reactions within the bulk ice. Thus, the three primary innovations of this work are (i) the first astrochemical kinetic model to include cations, anions, and free electrons as persistent species within the bulk ice mantle, allowing them to undergo nondiffusive ion–neutral, ion–ion, and dissociative recombination reactions in the solid phase. (ii) two new kinetic parameters to define the rate coefficients of the additional ion-neutral and ion–ion reactions, extending the nondiffusive rate formalism of Shingledecker et al.^21^ to charged species; and (iii) the first computational study of secondary ion chemistry in a UV-irradiated cosmic ice analogue. We note, regarding point (i), that this is distinct from recent models treating Eley–Rideal reactions of gas-phase ions with ice surfaces,^28^ which do not include internally generated bulk ionic species. Our paper is laid out as follows: In Section 2 we describe our theoretical and computational approach, in Section 3 we present the results of our trials and describe their astrochemical significance, and finally, we summarize our findings in Section 4.

## 2 Theory

### 2.1 Formation of Ions Within Cosmic Ice

Here, we are focused on the chemical contribution of ions produced within cosmic ice. In dense molecular clouds, as noted in the introduction, there exist two main mechanisms which could drive the ionization of dust-grain ice mantle species; namely, galactic cosmic rays^29,42^ and the UV photons they generate through the excitation of H_2_.^32^ The basis of the theoretical approach used in our models is that of Shingledecker and Herbst,^36^ which begins with the assumption of a set of four possible outcomes when some ice constituent, A, encounters some particle of ionizing radiation

R1 $$\mathrm{aA}⇝aA^{+}+e^{-}$$

R2 $$\mathrm{aA}⇝aA^{+}+e^{-}\to aA^{*}\to bB^{*}+cC^{*}$$

R3 $$\mathrm{aA}⇝aA^{*}\to \mathrm{bB}+\mathrm{cC}$$

R4 $$\mathrm{aA}⇝aA^{*}$$

where the lower case letters represent the relevant stoichiometric coefficients. Processes ([Disp-formula eq1]) and ([Disp-formula eq2]) correspond to the ionization of A, while ([Disp-formula eq3]) and ([Disp-formula eq4]) represent the effects of excitation to a higher vibrational or electronic state. Shingledecker and Herbst^36^ furthermore derive expressions for the rate coefficients ([Disp-formula eq1])–([Disp-formula eq4]), albeit for cases where A encounters a particle such as an energetic proton. This formalism was later expanded by Shingledecker et al.^34^ to treat irradiation by photons.

In large part because grain-chemical networks have only included neutral species, previous modeling works using one of the above approaches have made the assumption that in every case, the ionization of A proceeds via ([Disp-formula eq2]) rather than ([Disp-formula eq1]), i.e., that ionization is followed by rapid charge recombination to yield some excited suprathermal products. In contrast, here we assume as a first-order approximation that the two ionization processes, ([Disp-formula eq1]) and ([Disp-formula eq2]), occur with equal probability.

Briefly, to obtain rate coefficients (*k*_photo_) for ([Disp-formula eq1])–([Disp-formula eq4]), we begin with the standard formula for photoprocesses

1 $$k_{\mathrm{photo}}=\int \sigma \left(\lambda \right)I\left(\lambda \right)d\lambda$$

where the resulting value is a function of the wavelength-dependent absorption cross sections (σ­(λ)) and intensities (*I*(λ)) integrated over all wavelengths.^43^ To obtain the ultimately utilized expression for the rate coefficients, we express eq 5 as

2 $$k_{\mathrm{photo}}=\frac{\int \sigma \left(\lambda \right)I\left(\lambda \right)d\lambda }{\int I\left(\lambda \right)d\lambda }\int I\left(\lambda \right)d\lambda =\mathrm{\sigma ̅}\Phi$$

such that the rate coefficient can be written as the product of an average cross section for a given species and the photon flux integrated over all wavelengths.

As described in Mullikin et al.,^34^ the individual *k*_photo_ values for ([Disp-formula eq1])–([Disp-formula eq4]) are then multiplied by some branching fractions, *f*_br_. For a given species, we divide the branching fractions such that all ionization processes for that species add to unity (Type R1 and R2 in Table 1), and all excitation processes (R3 and R4) for a given species likewise add to unity.

**Table 1:** Photoprocesses Included in Our Model

number	type	process	*f*_br_	σ̅ (cm^2^)
P1	R1	$O⇝O^{+}+e^{-}$	0.50	0.00
P2	R1	$O_{2}⇝O^{+}+O^{-}$	0.25	3.86 × 10^–20^
P3	R1	$O_{2}⇝O_{2}^{+}+e^{-}$	0.25	3.86 × 10^–20^
P4	R1	$O_{3}⇝O^{+}+O_{2}^{-}$	0.167	0.00
P5	R1	$O_{3}⇝O_{2}^{+}+O^{-}$	0.167	0.00
P6	R1	$O_{3}⇝O_{3}^{+}+e^{-}$	0.167	0.00
P7	R2	$O⇝O^{*}$	0.50	0.00
P8	R2	$O_{2}⇝O^{*}+O^{*}$	0.50	3.86 × 10
P9	R2	$O_{3}⇝O_{2}^{*}+O^{*}$	0.50	0.00
P10	R3	$O_{2}⇝O+O$	0.50	2.13 × 10^–18^
P11	R3	$O_{3}⇝O_{2}+O$	0.50	5.60 × 10^–18^
P12	R4	$O⇝O^{*}$	1.00	0.00
P13	R4	$O_{2}⇝O_{2}^{*}$	0.50	2.13 × 10^–18^
P14	R4	$O_{3}⇝O_{3}^{*}$	0.50	5.60 × 10^–18^

Mullikin et al. also introduces the factor δ, which accounts for any discrepancy due to unavailable data, such as the difference between the actual solid phase cross sections compared with the gas-phase values used here. Thus, the rate coefficients for photodriven processes in our model are given by

3 $$k_{\mathrm{photo}}=f_{\mathrm{br}}\mathrm{\sigma ̅}\Phi \delta$$

Shown in Table 1 are the cross sections and branching fractions for the various processes used here, taken from Mullikin et al.^34^ Each separate process in that table is given a label prefixed with “P” while the generic type of process using the four pathways given above and described in Shingledecker et al.^44^ are denoted R1 to R4. For ionization processes, the total branching fraction is split between pathway types R1 and R2, where applicable. Likewise for excitation, branching fractions are split between R3 and R4. Though gas-phase molecular oxygen has an ionization energy of ∼12 eV, slightly higher than the UV photon energies used by Gerakines et al.,^41^ these values are typically lowered by a few eV in solids, leading to a nonzero cross section for processes P2 and P3. We note that processes P7 and P12 share the same net stoichiometry $\left(O⇝O^{*}\right)$ but represent fundamentally distinct physical mechanisms with different rate expressions. P7 is an R2-type process (ionization of O followed immediately by charge recombination), whose rate is proportional to the ionization cross section of O; since the ionization potential of atomic oxygen (13.6 eV) exceeds the maximum photon energy employed here, this cross section is effectively zero. P12 is an R4-type process (direct electronic excitation of O without ionization), which in principle carries a nonzero excitation cross section. The identical net reaction therefore reflects two distinct physical channels, not a duplication.

### 2.2 The Chemistry of Solid-Phase Ions

Thus formed within the ice as described in Section 2.1, the ions in our simulations are allowed to react chemically. Whether, or by which mechanism, reactions occur in low-temperature cosmic ice is still a matter of ongoing investigation. Here we assume, as in Shingledecker et al.,^21^ that rapid reactions can and do occur between neighbors even at temperatures as low as 5 K, in other words, nondiffusively. This approach was motivated by a now large body of experimental studies showing persuasively that even complex organic molecules (COMs) can be formed via fast solid-phase nondiffusive reactions^22,23,45–48^ and, when incorporated into simulations of astrophysical environments, yield predicted abundances of ice-mantle species that have proven to be in general agreement with recent JWST observations.^49–51^

As described in Shingledecker et al.,^21^ the fast nondiffusive bulk rate coefficient for some reaction A + B is given by

4 $$k_{\mathrm{fast}}=f_{\mathrm{br}}\left[\frac{\nu_{0}^{A}+\nu_{0}^{B}}{n_{\mathrm{bulk}}}\right]\exp\left(-\frac{E_{\mathrm{act}}^{\mathrm{AB}}}{T_{\mathrm{ice}}}\right)$$

where, ν_0_^A^ and ν_0_^B^ are the vibrational frequencies of A and B, *n*_bulk_ is the number of species in the ice mantle, exclusive of the surface layers, *E*_act_ is the activation energy in units of Kelvin, if present, and *T*_ice_ is the ice temperature. If one of the vibrational frequencies is much larger than the other, for example where ν_0_^A^ ≫ ν_0_^B^, then eq 8 can be simplified to

5 $$k_{\mathrm{fast}}\approx f_{\mathrm{br}}\left[\frac{\nu_{n-n}}{n_{\mathrm{bulk}}}\right]\exp\left(-\frac{E_{\mathrm{act}}^{\mathrm{AB}}}{T_{\mathrm{ice}}}\right)$$

where ν_n–n_ = ν_0_^A^. Hereafter, we will refer to ν_n–n_ as the neutral–neutral trial frequency.

As originally described in Shingledecker et al.,^21^ the species undergoing fast solid-phase reactions were all neutrals. For the purposes of this work, we have modified eq 8 slightly to also treat the solid-phase reaction of ions. First, for reactions between ions and neutrals of the forms A^+^ + B and A + B^–^, we replace ν_n–n_ with another frequency ν_i–n_, the ion-neutral trial frequency. Finally, for reactions between two charged species of the form A^+^ + B^–^, we invoke a third frequency, ν_i–i_, the ion–ion trial frequency. Here, the anions do not form directly from any of the processes in Table 1, but rather, result from, e.g., electron attachment reactions. The three frequencies in eq 9 represent the different long-range forces experienced between the reactants and account for the potentially higher overall reaction rate between a cation and an anion relative to a reaction involving two neutral species, even in an environment where diffusion is severely inhibited. As we describe in Section 3, these three frequencies were varied in our models to ascertain their influence on the resulting predicted abundances. As noted by Theulé et al.,^19^ the order of magnitude of the pre-exponential frequencies can vary widely, depending on the underlying mechanism and phase where reactions are occurring, e.g., gas or liquid. The same work further notes an enormous possible range of values for first-order processes between 10^13^ s^–1^ in the gas phase to as low as ∼10^–3^ s^–1^ in other cases, with those in condensed media being lower. The underlying causes for such variation are not, as far as we know, currently understood but could be related to effects such as the entropy of activation. Physically, such variation could reflect the strong configurational constraints imposed by the condensed-phase environment. In the language of transition-state-theory, this could manifest as an effective entropy-of-activation effect, though we do not regard that explanation as unique here.^19^

### 2.3 Model & Chemical Network

#### 2.3.1 Reaction Network

The reaction network in this work was limited to oxygen species only, since it is arguably the simplest radiation-chemical system, and for which there exist relevant experimental data in the work of Gerakines et al.^41^ We note that a number of additional experimental studies of photolysis and radiolysis of pure oxygen ice are relevant to the broader context of this network. In particular, Zhen and Linnartz^52^ report UV-photodesorption and photochemistry cross sections for O_2_ ice, and Sivaraman et al.^53^ examine the temperature-dependent formation of ozone under 5 keV electron irradiation; the ozone yields reported in both works are qualitatively consistent with the photochemical pathways included in our model, and both works are in broad agreement with our findings, described below, regarding the dominant chemical pathways to ozone formation. The starting point for our network was the oxygen network in Mullikin et al.,^34^ shown in Table 2, which in turn was based on previous work by Shingledecker et al.^21^ and Shingledecker et al.^44^ Our base network,^34^ which included only reactions between neutral species, was expanded with the reactions shown in Table 3 to include charge recombination reactions of the form

6 $$A^{+}+B^{-}\to C+D$$

using the reaction networks in Pastina and LaVerne,^54^ Anicich,^55^ and the NIST Chemical WebBook[Fn fn1]. Beyond extending ion-neutral reactions beyond the gas-phase and into our grain-chemical network, we have further added two more classes of reactions involving two species with unlike charges; namely, ion–ion recombination of the form

7 $$A^{+}+B^{-}\to C^{*}+D^{*}$$

and dissociative recombination between cations and electrons of the form

8 $$A^{+}+e^{-}\to C^{*}+D^{*}$$

**Table 2:** Base Chemical Network Used to Model Solid-Phase O_2_ Photo-Processing and Subsequent Chemistry, Taken from Mullikin et al.^34^ and Shingledecker et al.^21^

non-photon-induced reactions		
$O_{3}^{*}+O_{3}\to O_{2}+O_{2}+O_{2}$	$O_{2}^{*}+O\to O_{3}^{*}$	$O^{*}+O_{3}^{*}\to O_{2}+O_{2}$
$O+O\to O_{2}^{*}$	$O_{2}^{*}+O\to O_{2}+O$	$O_{2}^{*}+O_{2}^{*}\to O_{2}+O_{2}$
$O^{*}+O\to O+O$	$O_{2}^{*}+O_{2}\to O_{2}+O_{2}$	$O_{2}^{*}+O_{3}^{*}\to O_{2}+O_{2}+O$
$O^{*}+O\to O_{2}^{*}$	$O_{2}^{*}+O_{3}\to O_{2}+O_{2}+O$	$O_{3}^{*}+O_{3}^{*}\to O_{2}+O_{2}+O_{2}$
$O^{*}+O_{2}\to O_{3}^{*}$	$O^{*}+O^{*}\to O+O$	$O+O_{3}^{*}\to O_{2}+O_{2}$
$O^{*}+O_{2}\to O+O_{2}$	$O^{*}+O_{2}^{*}\to O_{3}^{*}$	$O_{2}+O_{3}^{*}\to O_{2}+O_{2}+O$
$O+O_{3}\to O_{2}+O_{2}$	$O^{*}+O_{2}^{*}\to O+O_{2}$	$O+O_{2}\to O_{3}^{*}$
$$O^{*}+O_{3}\to O_{2}+O_{2}$$		

**Table 3:** New Reactions Involving Charged Species Added for This Work

number	reaction	branching fraction
N1	$O+e^{-}\to O^{-}$	1.00
N2	$O+O^{-}\to O_{2}^{-}$	1.00
N3	$O+O_{2}^{-}\to O_{3}^{-}$	1.00
N4	$O^{+}+e^{-}\to O^{*}$	1.00
N5	$O^{+}+O^{-}\to O_{2}^{*}$	0.50
N6	$O^{+}+O^{-}\to O^{*}+O^{*}$	0.50
N7	$O^{+}+O_{2}\to O_{2}^{+}+O$	1.00
N8	$O^{+}+O_{2}^{-}\to O_{3}^{*}$	0.50
N9	$O^{+}+O_{2}^{-}\to O^{*}+O_{2}^{*}$	0.50
N10	$O^{+}+O_{3}^{-}\to O^{*}+O_{3}^{*}$	0.50
N11	$O^{+}+O_{3}^{-}\to O_{2}^{*}+O_{2}^{*}$	0.50
N12	$O_{2}+e^{-}\to O_{2}^{-}$	1.00
N13	$O_{2}+O^{-}\to O_{3}^{-}$	1.00
N14	$O_{2}^{+}+e^{-}\to O_{2}^{*}$	0.50
N15	$O_{2}^{+}+e^{-}\to O^{*}+O^{*}$	0.50
N16	$O_{2}^{+}+O^{-}\to O_{3}^{*}$	0.50
N17	$O_{2}^{+}+O^{-}\to O^{*}+O_{2}^{*}$	0.50
N18	$O_{2}^{+}+O_{2}\to O_{3}^{+}+O$	1.00
N19	$O_{2}^{+}+O_{2}^{-}\to O^{*}+O_{3}^{*}$	0.50
N20	$O_{2}^{+}+O_{2}^{-}\to O_{2}^{*}+O_{2}^{*}$	0.50
N21	$O_{2}^{+}+O_{3}^{-}\to O_{2}^{*}+O_{3}^{*}$	0.33
N22	$O_{2}^{+}+O_{3}^{-}\to O_{2}^{*}+O_{2}^{*}+O^{*}$	0.33
N23	$O_{2}^{+}+O_{3}^{-}\to O_{3}^{*}+O^{*}+O^{*}$	0.33
N24	$O_{3}+e^{-}\to O_{3}^{-}$	1.00
N25	$O_{3}+O^{-}\to O_{2}^{-}+O_{2}$	1.00
N26	$O_{3}+O_{2}^{-}\to O_{3}^{-}+O_{2}$	1.00
N27	$O_{3}+O_{3}^{+}\to O_{2}+O_{2}+O_{2}^{+}$	1.00
N28	$O_{3}^{-}+O^{-}\to O_{2}^{-}+O_{2}^{-}$	1.00
N29	$O_{3}^{+}+e^{-}\to O_{3}^{*}$	0.50
N30	$O_{3}^{+}+e^{-}\to O_{2}^{*}+O^{*}$	0.50
N31	$O_{3}^{+}+O^{-}\to O^{*}+O_{3}^{*}$	0.50
N32	$O_{3}^{+}+O^{-}\to O_{2}^{*}+O_{2}^{*}$	0.50
N33	$O_{3}^{+}+O_{2}\to O_{2}^{+}+O_{3}$	0.415
N34	$O_{3}^{+}+O_{2}\to O_{3}^{+}+O_{2}$	0.085
N35	$O_{3}^{+}+O_{2}\to O_{2}+O_{2}+O^{+}$	0.50
N36	$O_{3}^{+}+O_{2}^{-}\to O_{2}^{*}+O_{3}^{*}$	0.33
N37	$O_{3}^{+}+O_{2}^{-}\to O_{2}^{*}+O_{2}^{*}+O^{*}$	0.33
N38	$O_{3}^{+}+O_{2}^{-}\to O_{3}^{*}+O^{*}+O^{*}$	0.33
N39	$O_{3}^{+}+O_{3}^{-}\to O_{3}^{*}+O_{3}^{*}$	0.33
N40	$O_{3}^{+}+O_{3}^{-}\to O_{3}^{*}+O_{2}^{*}+O^{*}$	0.33
N41	$O_{3}^{+}+O_{3}^{-}\to O_{2}^{*}+O_{2}^{*}+O_{2}^{*}$	0.33

In both of the two cases above, we assume that the products are created in an excited suprathermal state, given the high exothermicity of the process. All new reactions involving ions were assumed to proceed barrierlessly. These ion-induced reactions enter the standard rate-equation formalism as additional formation and destruction terms, with rate coefficients given by eq 9 using the ionic trial frequencies ν*_i–n_* or ν*_i_*_–_ *_i_* as appropriate, and with the assumption of *E*_act_ = 0 for all charged-species reactions. UV photolysis is included explicitly via the photoprocess rate coefficients in Table 1, which compete with all chemical reactions at each time step. Thermal diffusion is deliberately excluded: our nondiffusive framework (Section 2.2) is motivated by experimental evidence that bulk ice reactions can occur even at temperatures where thermal diffusion is negligible. Grain surface reactions are included, but should be viewed with somewhat more skepticism, since our comparison here is with data relevant for bulk chemistry. We also note that the present model assumes spatially uniform (homogeneous) chemical processing throughout the ice mantle, which is a fundamental limitation of standard rate-equation astrochemical models, though the region over which abundances are averaged can be subdivided as in the multilayer model of Taquet et al.^56^ and Vasyunin et al.,^57^ yielding greater physical realism in terms of ice composition. In the context of UV-photon irradiation, where energy is deposited relatively uniformly over the penetration depth of thin ice films, this assumption is better justified than it would be for MeV heavy-ion bombardment, where spatial inhomogeneity along ion tracks can be significant. Sputtering, compaction, and structural modifications of the ice are not modeled in this work, though those processes are natural future extensions of this work.

### 2.4 Model Parameters

In order to examine the role of condensed-phase ion–molecule reactions under well-constrained conditions, we have simulated previous laboratory experiments directly, rather than an interstellar region. Specifically, we have sought to reproduce the experiments reported in Gerakines et al.,^41^ which studied the effects of irradiating a pure molecular oxygen ice deposited on a MgF_2_ substrate at a temperature of 10 K with UV photons (<11.3 eV) from a microwave-discharge hydrogen lamp (MDHL) with a photon flux of 2.2 × 10^14^ photons cm^–2^ s^–1^ as reported in Gerakines et al.^41^ Though this is a direct simulation of an experiment, our method could be easily extended to real interstellar sources via, e.g., adjusting the flux to an appropriate ISM value.

Here, a suite of simulations were carried out to simulate this ice irradiation using a variety of kinetic parameters, with the goal of understanding the dependence of the underlying chemistry on these values. A full list of the models run, and the values of the varied parameters, is given in Table 4.

**Table 4:** Model Sets Described Here, and Their Associated Parameters

model	ν_n–n_ (s^–1^)	ν_i–n_ (s^–1^)	ν_i–i_ (s^–1^)
1	5.00 × 10^8^	3.01 × 10^7^	6.93 × 10^11^
2	1.00 × 10^13^	1.00 × 10^13^	1.00 × 10^13^
3	1.00 × 10^13^	1.00 × 10^13^	1.00 × 10^10^
4	1.00 × 10^13^	1.00 × 10^13^	1.00 × 10^8^
5	1.00 × 10^13^	1.00 × 10^10^	1.00 × 10^13^
6	1.00 × 10^13^	1.00 × 10^10^	1.00 × 10^10^
7	1.00 × 10^13^	1.00 × 10^10^	1.00 × 10^8^
8	1.00 × 10^13^	1.00 × 10^8^	1.00 × 10^13^
9	1.00 × 10^13^	1.00 × 10^8^	1.00 × 10^10^
10	1.00 × 10^13^	1.00 × 10^8^	1.00 × 10^8^
11	1.00 × 10^10^	1.00 × 10^13^	1.00 × 10^13^
12	1.00 × 10^10^	1.00 × 10^13^	1.00 × 10^10^
13	1.00 × 10^10^	1.00 × 10^13^	1.00 × 10^8^
14	1.00 × 10^10^	1.00 × 10^10^	1.00 × 10^13^
15	1.00 × 10^10^	1.00 × 10^10^	1.00 × 10^10^
16	1.00 × 10^10^	1.00 × 10^10^	1.00 × 10^8^
17	1.00 × 10^10^	1.00 × 10^8^	1.00 × 10^13^
18	1.00 × 10^10^	1.00 × 10^8^	1.00 × 10^10^
19	1.00 × 10^10^	1.00 × 10^8^	1.00 × 10^8^
20	1.00 × 10^8^	1.00 × 10^13^	1.00 × 10^13^
21	1.00 × 10^8^	1.00 × 10^13^	1.00 × 10^10^
22	1.00 × 10^8^	1.00 × 10^13^	1.00 × 10^8^
23	1.00 × 10^8^	1.00 × 10^10^	1.00 × 10^13^
24	1.00 × 10^8^	1.00 × 10^10^	1.00 × 10^10^
25	1.00 × 10^8^	1.00 × 10^10^	1.00 × 10^8^
26	1.00 × 10^8^	1.00 × 10^8^	1.00 × 10^13^
27	1.00 × 10^8^	1.00 × 10^8^	1.00 × 10^10^
28	1.00 × 10^8^	1.00 × 10^8^	1.00 × 10^8^

For Model 1, we used a method for achieving optimal agreement between calculated and experimental data based on the approach used in Mullikin et al.^34^ The three aforementioned trial frequencies, ν_n–n_, ν_i–n_, and ν_i–i_, as well as the δ factors for select reactions described below were allowed to vary. The overall agreement between a given model and the experimental data was characterized with the value, χ, given by

9 $$\chi =\sqrt{\frac{\sum_{i=1}^{n}{\left(m_{i}-e_{i}\right)}^{2}}{n}}$$

where *e*_*i*_ and *m*_*i*_ are the *i*th experimental and model data points out of a total of *n*.

Regarding the variation of δ values, the results of a sensitivity analysis carried out by us found that calculated O_3_ abundances were sensitive to processes with a nonzero cross section, σ̅, excepting P10.

## 3 Results and Discussion

### 3.1 Best-Fit Model

In Table 5, we show values for the δ parameters for each photo process that yielded the best fit to experimental data. Shown in Figure 1 are the calculated abundances for O_3_ vs fluence for Model 1. Here, the fluence is defined as the product of the flux and the time. In Model 1, which resulted in χ = 0.484, the value of ν_i–i_ is set to a value of ∼7 × 10^11^ s^–1^, several orders of magnitude higher than the other frequency values of ν_n–n_ = 5 × 10^8^ s^–1^ and ν_i–n_ = 3 × 10^7^ s^–1^. This comparatively high value of ν_*i*–*i*_, coupled with a low value for ν_i–n_, tends toward the limiting case where ions produced in the bulk recombine very quickly, corresponding to the assumption made in our prior work where solid-phase ions were not present in the bulk.^34,36,49^ Previously, we assumed that ionization proceeded solely via process ([Disp-formula eq2]). A comparison between the abundances of Model 1 here and the results described in Mullikin et al.^34^ shows broadly good agreement, as expected, though the exact parameters obtained differ somewhat due to the greatly expanded network. We note that Gerakines et al.^41^ reported a peak O_3_/O_2_ ratio of ∼36%, whereas the maximum ratio reached in Model 1 is ∼24%. However, as in Mullikin et al., we interpret this value as a percentage of the total initial oxygen atoms. Multiplying our final abundances by a factor of 1.5, to account for this, brings our results in exact agreement with the experimental value.

**Table 5:** Delta Value for Photoprocesses in Model 1

number	type	process	δ
P1	R1	$O⇝O^{+}+e^{-}$	1.00
P2	R1	$O_{2}⇝O^{+}+O^{-}$	0.437
P3	R1	$O_{2}⇝O_{2}^{+}+e^{-}$	1.54
P4	R1	$O_{3}⇝O^{+}+O_{2}^{-}$	1.00
P5	R1	$O_{3}⇝O_{2}^{+}+O^{-}$	1.00
P6	R1	$O_{3}⇝O_{3}^{+}+e^{-}$	1.00
P7	R2	$O⇝O^{*}$	1.00
P8	R2	$O_{2}⇝O^{*}+O^{*}$	1.90
P9	R2	$O_{3}⇝O_{2}^{*}+O^{*}$	1.00
P10	R3	$O_{2}⇝O+O$	1.00
P11	R3	$O_{3}⇝O_{2}+O$	22.1
P12	R4	$O⇝O^{*}$	1.00
P13	R4	$O_{2}⇝O_{2}^{*}$	99.0
P14	R4	$O_{3}⇝O_{3}^{*}$	99.0

**Figure 1:**
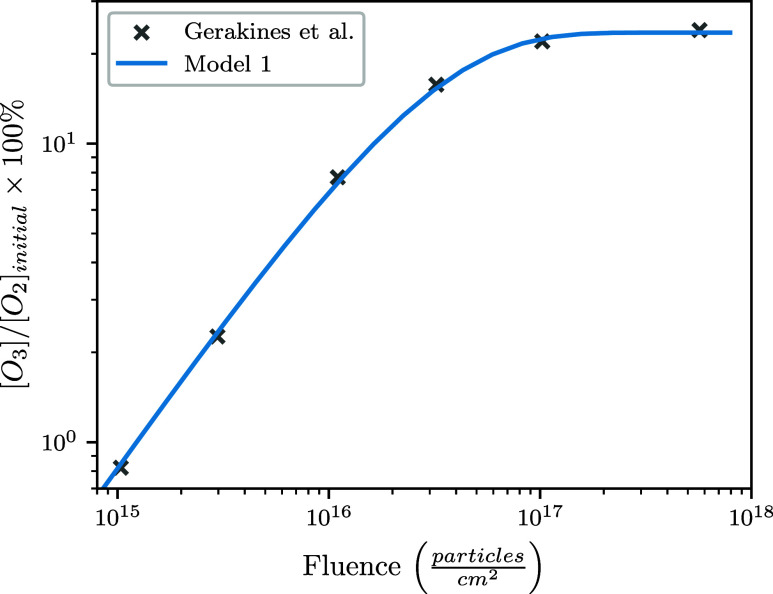
Model 1 calculated abundances of O_3_ in best-fit model compared with experimental data.

### 3.2 Other Models

As shown in Table 4, in addition to Model 1, our best-fit model, we also ran a number of other simulations to investigate the effects of key parameters on the resulting agreement with experimental data. The resulting ozone abundances are shown in Figure 2. In Models 2–28, all δ values were set to unity, while ν_n–n_, ν_i–n_, and ν_i–i_ were tested using all possible combinations of three different frequency values: low (1.00 × 10^8^ s^–1^), medium (1.00 × 10^10^ s^–1^), and high (1.00 × 10^13^ s^–1^). These models highlight the importance of the δ values in obtaining a good fit to the experimental data, and all of these test simulations resulted in values of χ > 12 denoting poor fit. In general, we found that models with higher relative values for ν_i–n_ gave the worst agreement, in line with the findings of our best-fit model. We also consistently found that, with our expanded chemical network, varying the values of ν_i–n_ and ν_i–i_ affected the calculated abundance of ozone much more than variations of ν_n–n_. This finding suggests that, though the dominant formation pathways of many of the species described below may be ultimately governed by neutral–neutral reactions, the included ionic chemistry had a significant effect on the abundances of those neutral species in our models.

**Figure 2:**
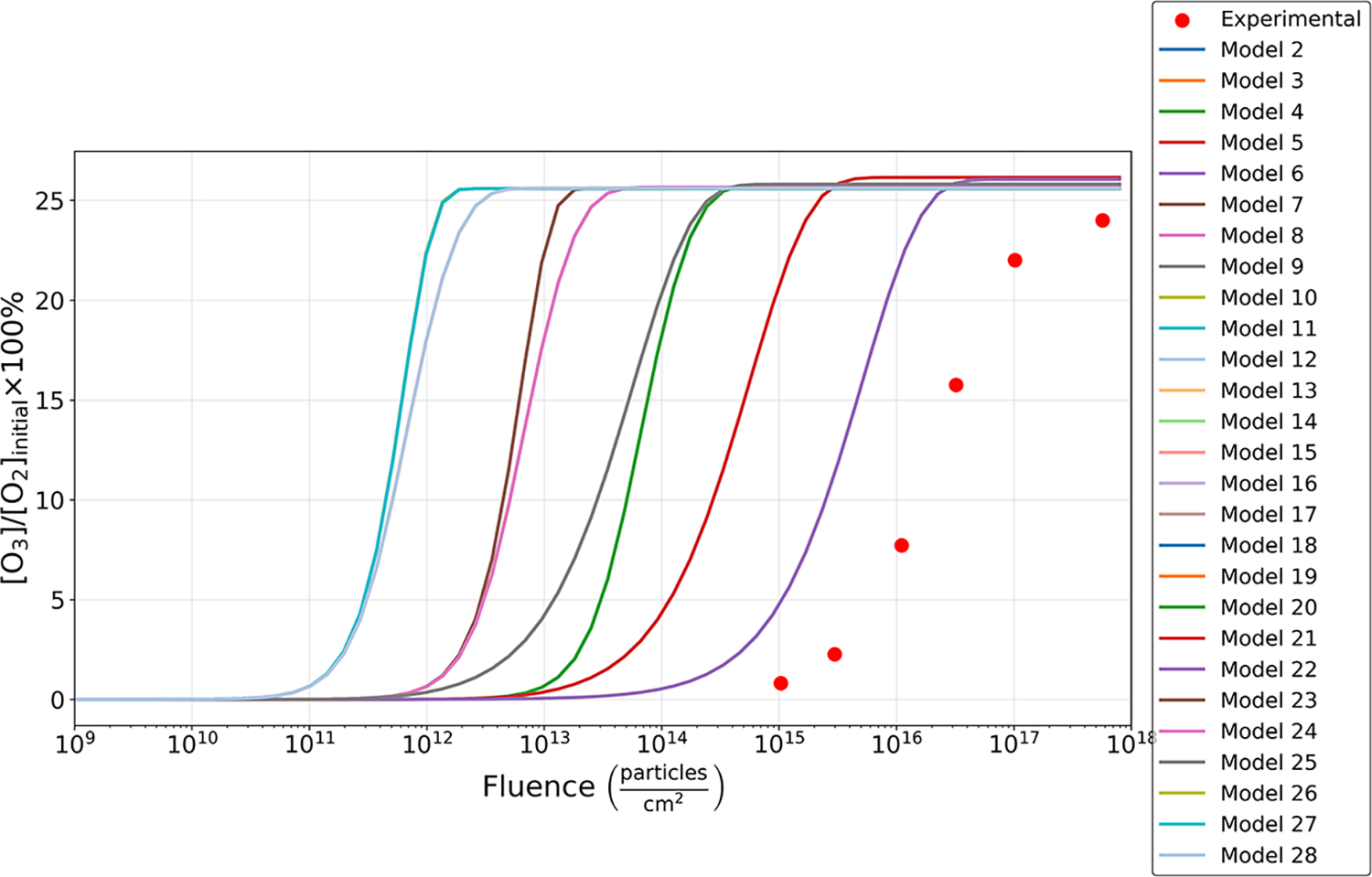
Models 2–28 (solid lines) compared with experimental data (points).

In the following sections, the chemistry of neutrals and ions is analyzed. We focus on the results of the best-fit Model 1.

### 3.3 Neutral Species

In this section, we detail the chemistry of the neutral species in our chemical network (O, O_2_, and O_3_), as well as of their suprathermal counterparts. Each of their major formation and destruction reactions is shown in Figure 3. We note that Table 6 describes the meaning of the different line styles in the reaction contribution figures presented here.

**Figure 3:**
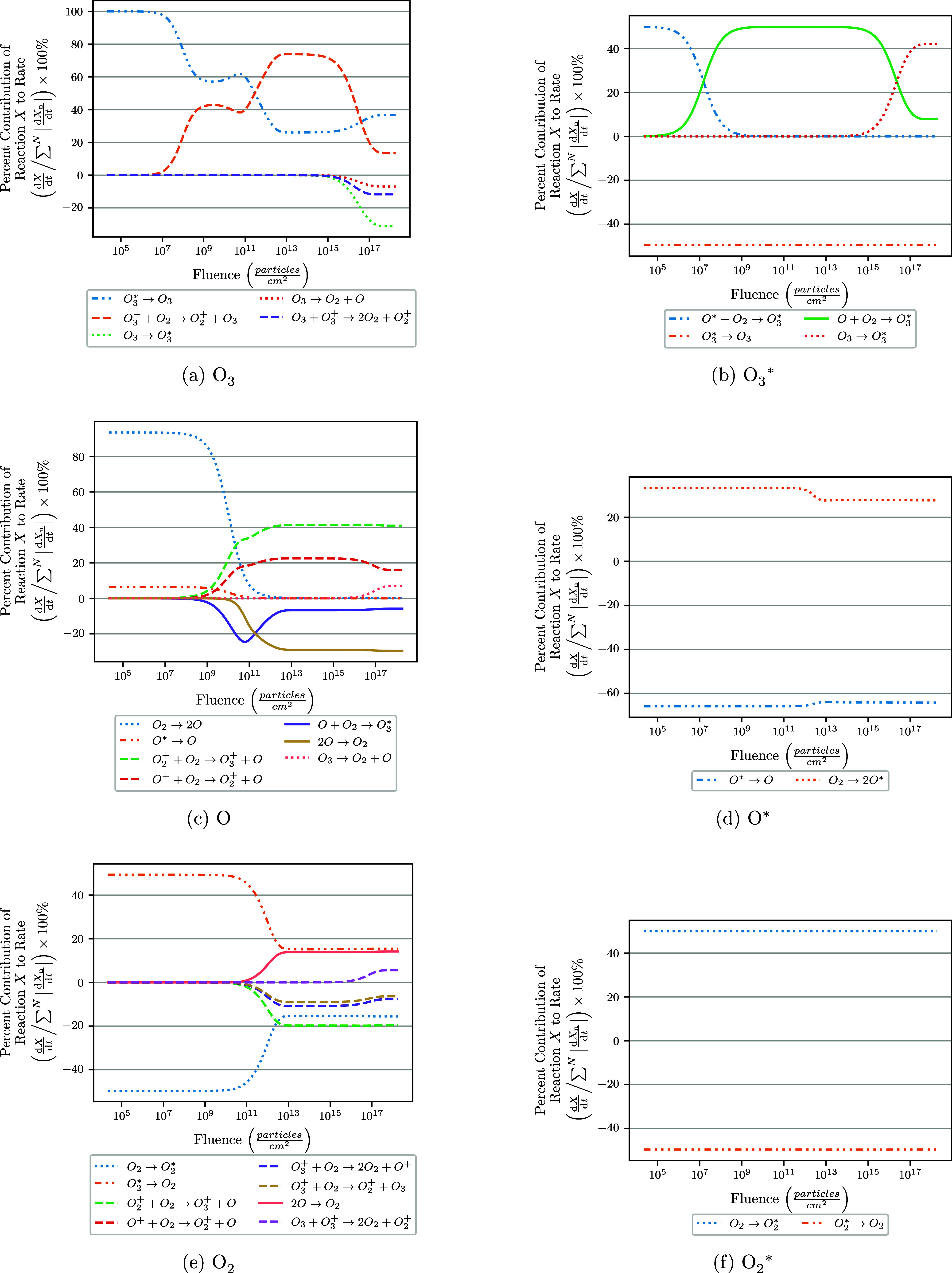
Relative importance of formation and destruction reactions for neutral species and their suprathermal counterparts in Model 1. Here, panel (a) shows O_3_, panel (b) shows O_3_*, panel (c) shows O, panel (d) shows O*, panel (e) shows O_2_, and panel (f) shows O_2_*. See Table 6 for an explanation of the different linestyles.

**Table 6:**
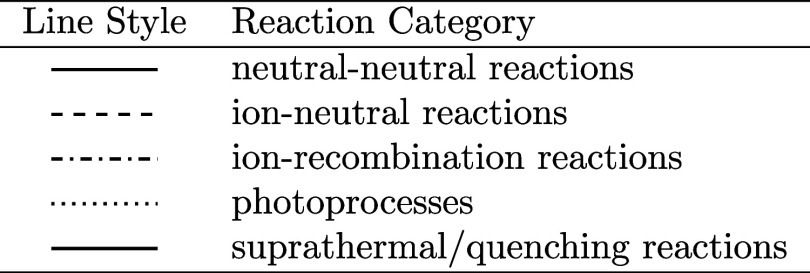
Line Styles in Reaction Contribution Plots

#### 3.3.1 O_3_ and O_3_ *

As shown in Figure 3a, the chemistry of ozone largely follows what was found previously in Mullikin et al.^34^ Here, as there, the dominant formation route for ozone is the two-step process

10 $$O+O_{2}\to O_{3}^{*}⇝O_{3}$$

where suprathermal O_3_* is first formed from the reaction of ground state atomic and molecular oxygen, or between suprathermal oxygen and ground state molecular oxygen. In both cases, suprathermal ozone is destroyed via quenching by the bulk ice (see Figure 3b).

Conversely, at intermediate fluences, the underlying chemistry of O_3_ in the model changes dramatically, highlighting the potentially critical role of ions for understanding the chemistry of irradiated low-temperature solids, with the dominant formation pathway (∼70%) becoming

N33 $$O_{3}^{+}+O_{2}\to O_{2}^{+}+O_{3}$$

In Model 1, ozone is destroyed mainly by either photolysis or reaction with O_3_^+^, again highlighting the potential importance of ions in bulk chemistry.

#### 3.3.2 O and O*

As can be seen in Figure 3c, the chemistry of atomic oxygen largely follows a similar pattern as O_3_. At early fluences, neutral chemistry dominates, with the main formation route being

P10 $$O_{2}⇝2O$$

and the major destruction pathways being the reformation of molecular oxygen

11 $$O+O\to O_{2}$$

or reaction with O_2_ to form suprathermal ozone.

However, as with O_3_, ion-neutral reactions become more important at later fluences, as depicted in Figure 3c. After a fluence of 10^11^ photons cm^–2^ is reached, the reactions

N18 $$O_{2}^{+}+O_{2}\to O_{3}^{+}+O$$

and

N7 $$O^{+}+O_{2}\to O_{2}^{+}+O$$

become more significant, representing roughly 40% and 20% of the O formation, respectively.

For the case of suprathermal oxygen, depicted in Figure 3d, the situation is quite simple. At all fluences, it is mainly formed via

P8 $$O_{2}⇝2O^{*}$$

Similarly, the major destruction pathway throughout the model is quenching by the ice.

#### 3.3.3 O_2_ and O_2_*

For O_2_ reactions, represented in Figure 3e, the dominant formation route at early fluences (before ∼ 10^12^ cm^–2^) comes simply from the quenching of suprathermal O_2_. After that time, the bimolecular reaction between ground-state oxygen atoms becomes more important. Destruction is initially dominated by electronic excitation leading to suprathermal molecular oxygen. After ∼ 10^12^ cm^–2^, ion-neutral destruction routes become more important, including the reaction of molecular oxygen with O_2_^+^ (N18), as well as the reactions

$$\begin{array}{lr} O_{3}^{+}+O_{2}\to O_{2}^{+}+O_{3} & \mathrm{(N33)} \end{array}$$

and

N35 $$O_{3}^{+}+O_{2}\to 2O_{2}+O^{+}$$

For suprathermal molecular oxygen, shown in Figure 3f, at all model times the dominant formation route is via the excitation process P13. Similar to the other suprathermal species, the dominant destruction pathway is via quenching.

### 3.4 Ions

Figure 4 shows the calculated abundances of the cations and anions in our simulation as a function of fluence. There, one can see that the combined peak abundance of all charged species remains very low, with a value of ∼4.6 × 10^–7^% at its peak. Here, we describe the main formation and destruction pathways of key ionic species.

**Figure 4:**
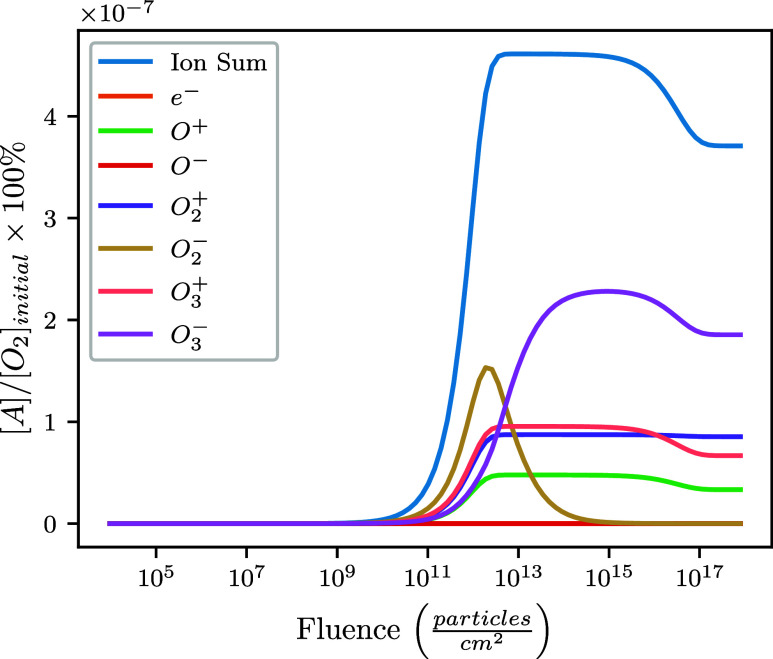
Total abundance of solid-phase ions vs fluence.

Before a fluence of ∼10^11^ cm^–2^, the abundance of ions is quite low. Afterward, however, there is a noticeable increase in their abundance, with O_2_^–^ being initially the most abundant. As depicted in Figure 5a, this anion is formed at all model times mainly via the associative attachment of an electron

N12 $$O_{2}+e^{-}\to O_{2}^{-}$$

and is destroyed via a number of reactions, with the most important after ∼10^13^ cm^–2^ being the charge transfer reaction

N26 $$O_{3}+O_{2}^{-}\to O_{2}+O_{3}^{-}$$

**Figure 5:**
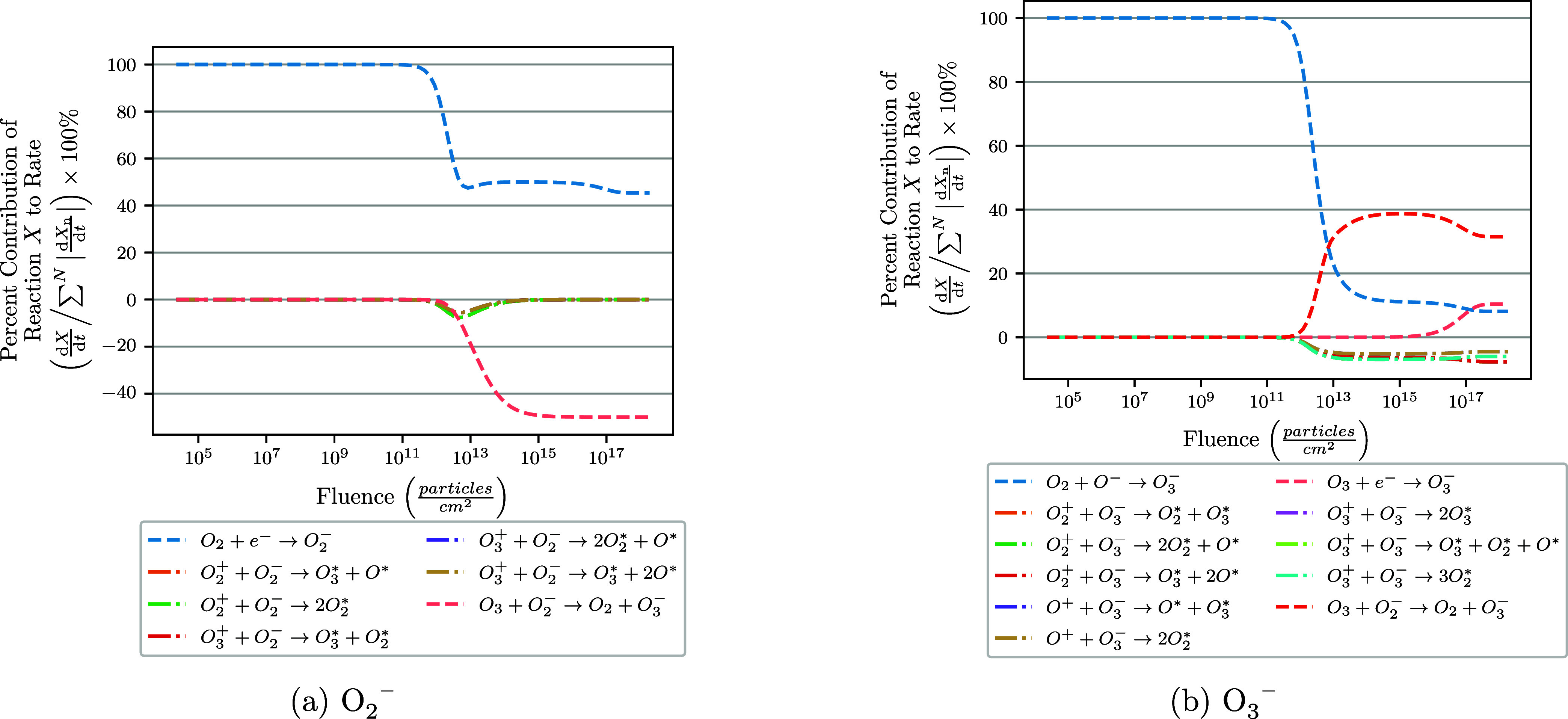
Relative importance of formation and destruction reactions for (a) O_2_^–^ and (b) O_3_^–^ in Model 1. See Table 6 for an explanation of the different linestyles.

After a fluence of ∼10^14^ cm^–2^, O_3_^–^ becomes the dominant charged species in Model 1. As shown in Figure 5b, it is initially formed mainly via

N13 $$O_{2}+O^{-}\to O_{3}^{-}$$

with the reaction

$$\begin{array}{lr} O_{3}+O_{2}^{-}\to O_{3}^{-}+O_{2} & \mathrm{(N26)} \end{array}$$

becoming important at later fluences. The destruction of O_3_^–^ occurs entirely through ion-recombination reactions. We note that the plots of the eight ion-recombination reactions obscure each other in Figure 5b.

#### 3.4.1 Electrons

Finally, the addition of electrons in our network is another novel feature and a byproduct of the ionizing radiation that real astrophysical ices are continuously exposed to. As shown in Figure 6, electron chemistry is dominated by the ionization and subsequent recombination with the dominant ice constituents. Specifically, they are produced overwhelmingly from the photoionization of O_2_ and destroyed via recombination with O_2_ and O_3_. We note that in these preliminary simulations, we have not included processes which are critical for very low energy (<5 eV) electrons, such as dissociative electron attachment (DEA), though such processes undoubtedly occur in real irradiated ices.^38^ Future simulations will examine the effects of low-energy electrons in more detail.

**Figure 6:**
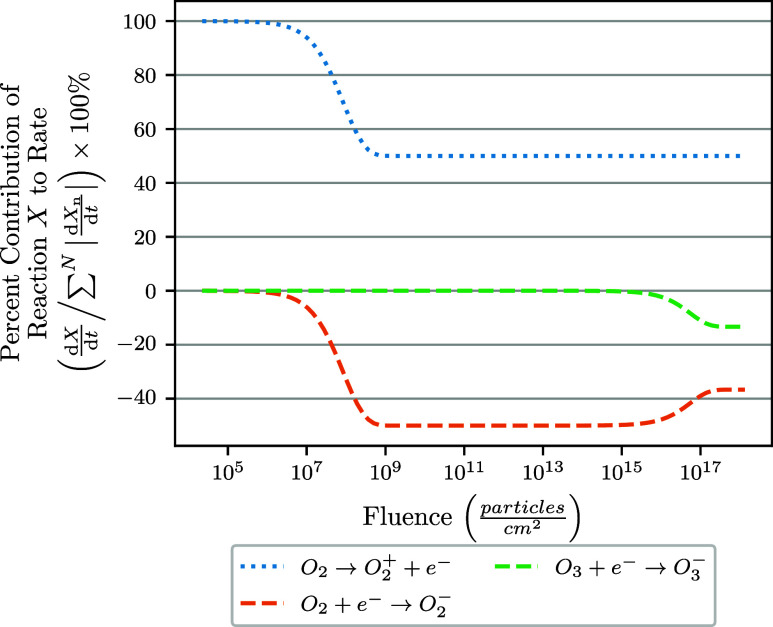
Major reactions with electrons.

### 3.5 The Impact of Ions in our Models

To directly assess the role of ionic channels in Model 1, we also carried out a control model in which only the photoprocess channels yielding charged products were suppressed, while all neutral channels, trial frequencies, and other Model 1 parameters were held fixed. In this control, agreement with the Gerakines et al.^41^ O_3_ data deteriorates markedly: the unweighted root-mean-square deviation χ increases from 0.484 (Model 1) to 13.93 in this control case, with the peak modeled O_3_ abundance falling from 24% to 3% of the initial O_2_. Calculated abundances for Model 1 and this control simulation are compared directly in Figure 7. Thus, while it remains the case that neutral–neutral reactions are the dominant formation routes for ozone in our new network, the overall impact of adding ionic reactions here is nontrivial on the overall chemistry, at least for the ion–optimized parameter set of Model 1.

**Figure 7:**
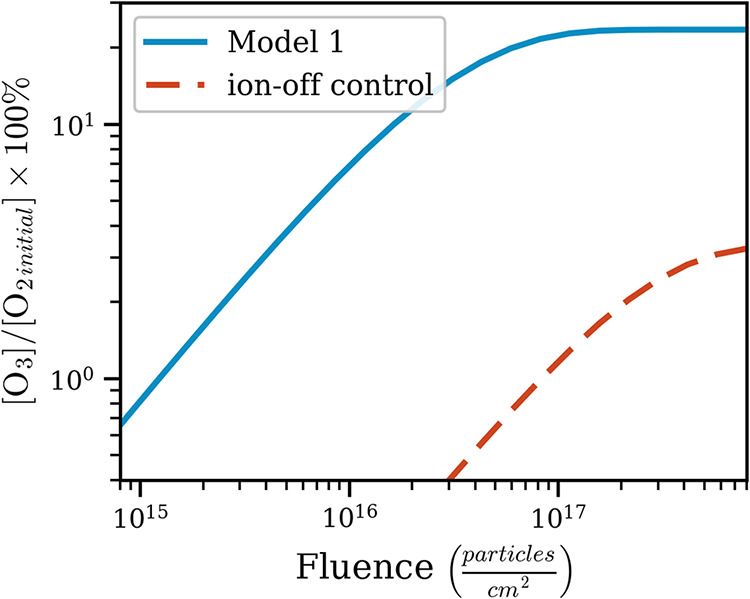
Calculated abundances for Model 1 (blue curve) and a control model using Model 1 input parameters, but with ion–forming photoprocesses disabled (red–dashed curve).

## 4 Conclusions

In this work, we describe novel astrochemical simulations which seek to elucidate the effects of solid-phase ions on the chemistry of low-temperature solids, specifically, within interstellar dust grain ice mantles. Expanding on our previous models described in Shingledecker et al.^21^ and Mullikin et al.,^34^ we have used the method of Shingledecker and Herbst^36^ to model the production of ionic species formed from the photoionization and photoexcitation of atoms and molecules within the ice. To examine the effects of such ions on solid-phase chemistry under well-constrained conditions, we carried out simulations of pure O_2_ ice at 10 K irradiated by UV photons, following the work of Gerakines et al.,^41^ who tracked the production of ozone under those conditions.

The models described here show that inclusion of ionic species and related reactions is possible in three-phase, slightly modified astrochemical codes, with the caveat that such additions do substantially increase the size, complexity, and uncertainty of the chemical networks. Our results further indicate that ionic pathways can be kinetically important for reproducing the observed O_3_ growth in Model 1. Though neutral channels generally dominate O_3_-forming steps, particularly at early and late fluence, ion-neutral and ion–ion processes become more important during midfluence chemistry. The stronger sensitivity of the calculated abundances to ν*_i–n_* and ν*_i–i_* than to ν*_n–n_* further suggests that, in this network, ionic reactions had a nontrivial role in regulating the neutral species abundances.

Future studies using this method could fruitfully be applied to studying systems where solid-phase ion-neutral and ion–ion reactions are likely to play a larger role, such as those involving the formation of salts or the kinds of charged species which have been detected in cometary material^58^ and real interstellar dust-grain ice mantles.^50,51^ Overall, though, the results of our calculations are in line with other recent studies, notably by Woon^24^ and Woon^37^ who studied bulk-ice chemistry, and both Nakai et al.^26^ and Cui and Herbst,^28^ who studied surface processes, in showing that, as is the case with gas-phase processes, the reactions of charged species on interstellar dust grains likely represent an important class of chemical processes that will need to be examined in more detail in the future. Of particular utility for future modeling studies would be experimental data on the fluence-dependent abundance of multiple components of an ice-analogue, as well as information about the total concentration and makeup of ions in situ in the ice.

## Footnotes

1
https://webbook.nist.gov/chemistry/
